# Complete genome sequence of the putative plant growth-promoting bacterium *Pseudomonas gorinensis* strain NG1 isolated from a high-lipid algae strain

**DOI:** 10.1128/mra.00330-26

**Published:** 2026-10-07

**Authors:** Elise K. Wilbourn, Brittany Humphrey, Mia Lobitz, J. Bryce Ricken, Chuck Smallwood, Jesse Cahill, Ryan W. Davis

**Affiliations:** 1 Bioresource and Environmental Security, Sandia National Laboratories 111651, Livermore, California, USA; 2 Environmental Systems Biology, Sandia National Laboratories 1105, Albuquerque, New Mexico, USA; University of Wisconsin-Madison, Madison, Wisconsin, USA

**Keywords:** *Pseudomonas*, algae

## Abstract

We report the genome sequence of *Pseudomonas gorinensis* NG1 isolated from alongside the alga *Nannochloropsis gaditana*. Bacteria capable of growing alongside algae cultured for biofuel production were isolated. The genome is 7,017,688 bp with 60.73% GC content and was sequenced with Oxford Nanopore technology and assembled with Flye.

## ANNOUNCEMENT

Algae-associated bacteria are of interest for their roles in crop protection (1) and algae growth promotion (2), especially in commercial algae crops grown in open-air ponds for biofuel production (3). *Pseudomonas gorinensis* NG1 was isolated from cultures of the green alga *Nannochloropsis gaditana* grown in sterile flasks containing f/2 media in a lighted incubator at Sandia National Laboratories (35.2147° N, 106.4506° W), which is of interest for its high lipid content (4). However, this alga is susceptible to various pests. This bacterium was isolated as part of a larger study to better characterize the roles of algae-associated microbiome members (4).

Bacteria were isolated from an algae-bacterial coculture cultivated in 80:20 vol:vol modified f/2 media:marine broth (Difco Marine Broth, BD Diagnostics) in 250 mL flasks in a lighted incubator (12 h diurnal cycle, 215 μmol m^−2^ s^−1^, 25°C). Bacteria were isolated from algae using a flow cytometer (BD FACSAria Fusion, Waters Bioscience). Colonies were streaked three times on Luria broth (LB) agar and incubated at 30°C overnight each round. Colonies were picked and resuspended in 20 μL water for PCR. Sanger sequencing used for initial 16S analysis was performed by Eton BioScience (San Diego, CA, USA). A fresh overnight culture was grown in LB overnight at 30°C, and 50 μL of culture was shipped overnight to Plasmidsaurus, Inc. (Louisville, KY, USA) for whole genome sequencing. Oxford Nanopore Technologies (ONT) long-read sequencing was performed by Plasmidsaurus, Inc. The library was prepared with the Ligation Sequencing Kit v14 and sequenced with the ONT PromethION R10.4.1 Flow Cell with the Dorado basecalling model using default Q10 quality filtering on super-accurate mode. Default parameters on FiltLong v0.2.1 were used to remove the worst 5% of reads.

The genome is 7,008,901 bp with 60.73% GC content and 121× coverage. The genome was assembled into one contig with an N50 value of 7,008,901 using Dragonflye v1.2.1 (https://github.com/rpetit3/dragonflye). Unless otherwise noted, default settings were used. The genome size was estimated with KMC v3.2.4 (5). Read length was filtered to 1,000 bp using nanoq v0.10.0 (6); adaptors were trimmed with Porechop v0.2.4 (https://github.com/rrwick/Porechop); and the genome was assembled with Flye v2.9.6 (7) through Dragonflye. The assembly was polished with Racon v1.5.0 (https://github.com/isovic/racon), and the circular genome was reoriented with dnaapler (8). The genome size and quality metrics were checked with CheckM2 v1.1.0 (9). CheckM2 showed a completeness of 100% and contamination of 2.56%. The genome was annotated with Prokka v1.15.6 (10). The largest proportion of the predicted proteins were annotated as hypothetical or putative proteins (2,436 out of 6,384 or 38%). The assembled genome was identified as *Pseudomonas gorinensis* using Kraken 2 with standard settings to search the full Silva database v138.2. The genome was determined to be 99.08% similar to *Pseudomonas gorinensis* NT1 using FastANI v1.0.0 (11).

## Data Availability

This Whole Genome Shotgun project has been deposited at DDBJ/ENA/GenBank under the accession number JBWBZN000000000, BioProject number PRJNA1436599, and BioSample number SAMN56475372. The version described in this paper is version JBWBZN000000000.1.
